# Graphene Oxide/BiOCl Nanocomposite Films as Efficient Visible Light Photocatalysts

**DOI:** 10.3389/fchem.2018.00274

**Published:** 2018-07-24

**Authors:** Weitian Lin, Xiang Yu, Yi Zhu, Yuanming Zhang

**Affiliations:** ^1^Department of Chemistry, Jinan University, Guangzhou, China; ^2^Analytical & Testing Center, Jinan University, Guangzhou, China

**Keywords:** graphene, BiOCl, film, visible-light, photocatalytic activity

## Abstract

A novel graphene oxide/BiOCl (GO/BiOCl) nanocomposite film was prepared *via* a spread coating method. In visible-light photocatalytically degrading Rhodamine B (RhB) experiments, 2 wt% GO/BiOCl could degrade 99% of RhB within 1.5 h and the rate constant was 12.2 times higher than that of pure BiOCl. The degradation efficiency still kept at 80% even after 4 recycles, evidencing the relatively good recyclability. The enhancement was attributed to the improvement of visible light adsorption and charge separation. Holes and superoxide radicals·${\text{O}}_{2}^{-}$ played a major role as reactive species. The values of conduction band and valence band for GO and BiOCl were calculated and a new photocatalytic mechanism of GO/BiOCl nanocomposite was proposed.

## Introduction

The problems of environmental pollution have been becoming a major concern with industrial development, especially water pollution, which severely impacts our lives (Maeda et al., 2006; Zhao et al., 2015; Nie et al., 2018). The photocatalysis technique is often used in dealing with water pollution because of its low cost, chemical stability, and non-toxicity (Dong et al., 2015; Meng and Zhang, 2016; Yang G. et al., 2017; Jing et al., 2018; Wu et al., 2018). The conventional UV-light-driven photocatalytic semiconducting materials, such as TiO_2_ and ZnO, can only be inspired by UV light accounting for <5% of sunlight (Liu et al., 2009; Yang et al., 2013; Chen et al., 2014a,b). Therefore, there is a need to exploit new visible-light photocatalysts with excellent photocatalytic performance. Among various photocatalytic materials, the bismuth compounds have attracted considerable attention for its relatively high photocatalytic activity (Li et al., 2014; Chen et al., 2018). As a V-VI-VII ternary semiconductor, bismuth oxychloxide (BiOCl) with a tetragonal crystal structure, consists of a tetragonal [Bi_2_O_2_]^2+^ positive slabs interleaved by double negative slabs of Cl atoms, which provides the space large enough to polarize the related atoms and orbits (Long et al., 2015; Li M. et al., 2017) and leads to its relatively high photocatalytic activity (Cheng et al., 2014). Nevertheless, BiOCl has been prevented by low separation of the photogenerated electron–hole pairs and UV-light-driving, which make it difficult for practical applications. So it is urgent to further improve electron–hole pairs separation and its visible light adsorption to achieve a high photocatalytic activity. Methods have been applied to change this situation, such as morphology control (Zhu et al., 2010), crystal facet exposure (Wang D. H. et al., 2012), noble metal doping (Jiang et al., 2013), and so on. Among them, fabricating nanocomposites by hybridizing BiOCl with other materials is a practical way. Li et al. showed that novel BiOI/BiOCl nanocomposite demonstrated notably high photocatalytic activities over methyl orange (MO) and RhB in aqueous solution. The enhanced photocatalytic activities for BiOI/BiOCl composites were ascribed to the matched conduction band and valence band level and effective separation of electron-hole pairs (Li et al., 2011). Wang et al. prepared the BiOCl-C_3_N_4_ heterojunction photocatalyst with high specific surface areas in a solvent-thermal way, which displayed notably high photocatalytic activity in decomposing MO under visible-light (Wang X. J. et al., 2013). Zhu and his coworkers reported N-doped carbon nanotube-BiOCl using a facile solvothermal method, exhibiting enhanced photocatalytic performance compared with pristine BiOCl for decomposition of RhB under UV-light (Zhu L. et al., 2016). Wang et al. successfully fabricated polyaniline/BiOCl photocatalysts with excellent visible-light photocatalytic activity toward MO. The enhancement of photocatalytic properties could be ascribed to the synergistic effect between BiOCl and polyaniline (Wang Q. et al., 2013).

Graphene, a new type of carbon material with monolayer of sp^2^-hybridized carbon atoms in honeycomb structure, has aroused great attention in electronic, optical, and catalytic fields due to its good conductivity, optical and electrical properties (Berger et al., 2006; Geim, 2009; Xie et al., 2015; Xiong et al., 2015; Zhu S. et al., 2016). Recently, graphene exhibits wide applications in photocatalysis including photocatalytic water-splitting to produce hydrogen and photo-degradation for organic pollutants because of its outstanding mobility of charge carriers and much higher theoretical specific surface area (Xiang et al., 2012a,b; Cao et al., 2014; Feng J. et al., 2017). Many studies have been focused on fabricating GO/semiconductor composites to achieve enhanced visible light photocatalytic activities (Yang et al., 2015). For example, Xu et al. reported an efficient graphene hybridized with ZnO photocatalyst for the improved UV light photocatalytic activity (Xu et al., 2011). Ai and her partners fabricated BiOBr-graphene nanocomposites and investigated the excellent visible-light photodegradation on gaseous nitrogen monoxide (Ai et al., 2011). Zhang et al. synthesized a P25-graphene nanocomposite using a facile one-step hydrothermal method and observed significant enhancement photocatalytic activities in degradation of MO (Zhang et al., 2010). Better photocatalytic performances may be obtained by combining remarkable properties of GO and BiOCl to form GO/BiOCl nanocomposites. Up to now, there have been only few investigations on graphene oxides (GO) or reduced graphene oxides (rGO)/BiOCl nanocomposites. Tian et al. prepared rGO-BiOCl hybrid materials *via* a facile solvothermal method. The 0.73% rGO-BiOCl hybrid showed the best photocatalytic degradation performance for RhB (Tian L. et al., 2013). Gao et al. reported chemically bonded graphene/BiOCl composites by a facile chemical-bath method, which exhibited the degradation rate twice as much as that of pure BiOCl over methylbenzene removal under UV-light (Gao et al., 2012). Kang et al. prepared size-controlled rGO-BiOCl with PVP using a hydrothermal method at low temperatures, which showed much higher visible-light photocatalytic activity toward RhB degradation, compared with pure BiOCl (Kang et al., 2015). But only GO/BiOCl nanocomposite powders were reported, which were hard to be separated and recycled. Synthesis of composite films is the most effective way to deal with these problems (Mu et al., 2012). However, there is no report on GO/BiOCl films, which are easy to be separated and recycled.

In our work, GO/BiOCl films were successfully fabricated by a spread coating method at room temperature. The prepared samples were characterized, and the photocatalytic properties were studied by degrading RhB under visible light irradiation. Accordingly, a new photocatalytic mechanism was also presented.

## Experimental

### Synthesis of GO/BiOCL

**Scheme 1:**
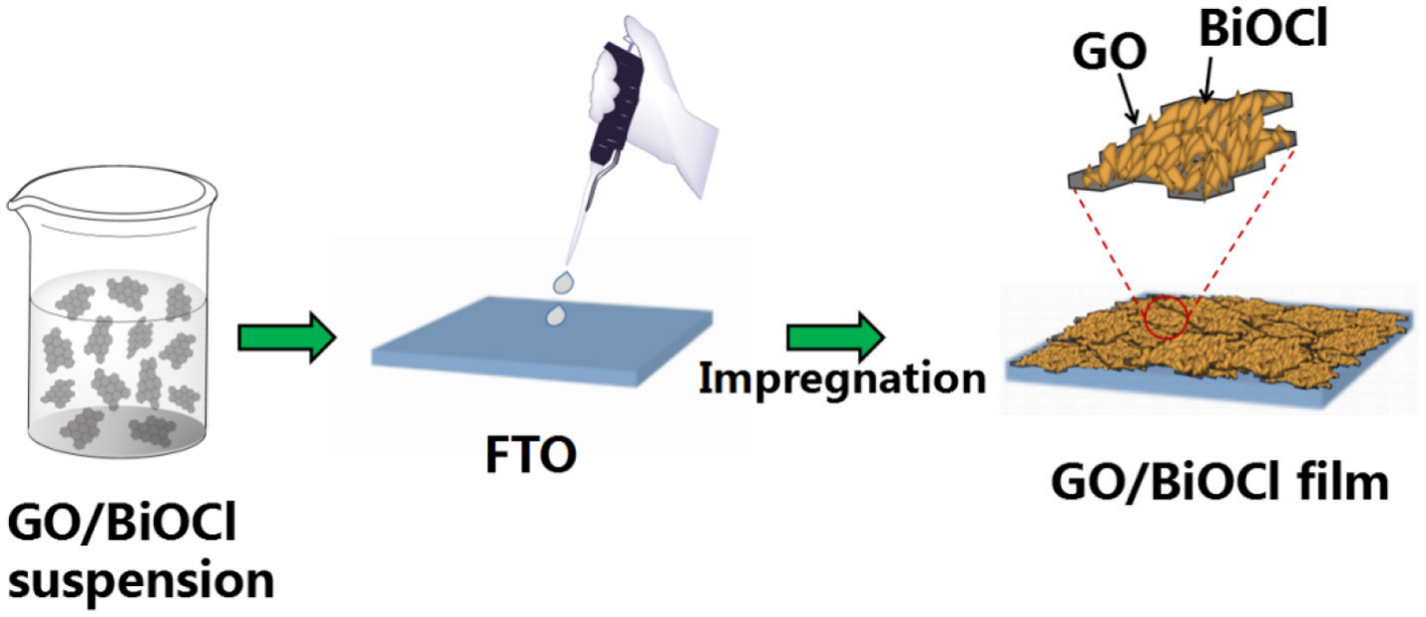
Schematic procedure for fabricating GO/BiOCl film.

The GO/BiOCl was synthesized on a FTO in a facile process. 1.5 g BiCl_3_ and 0.25 mL HCl were dissolved in 50 mL ethanol by ultrasonication for 30 min. After that, GO was added into the mixture, which was magnetically stirred at room temperature. Few milliliter of mixture was sucked and smeared homogeneously on FTO, and then dried at 80°C for 30 min. Finally, the FTO was immerged in distilled water, and then dried at 60°C. The approach of fabricating GO/BiOCl film was shown in Scheme 1. Different amounts (0.8, 2, 4, and 5 wt%) of GO were added. For comparison, BiOCl without GO was prepared in the similar way.

### Characterization

The structure and phase characterization of the as-prepared samples were recorded on an X-ray diffractometer (XD-2) at the scanning rate of 8°min^−1^. The morphologies of the samples were characterized by FE-SEM (Zeiss ULTRA 55) and FE-TEM (JEOL 2010F). EDS spectra were obtained using Bruker/Quanta 200 instrument. The ESCALab250 instrument was utilized to record the XPS spectra, which could be used to analyze the surface properties and chemical statement. The BET surface areas and N_2_ adsorption-desorption isotherms of the samples were investigated on a TriSTRA 3000 at 77.3 K. The light absorption of the samples was investigated with a UV-Vis diffuse reflectance spectrum (DRS, Hitachi UV-3010) with BaSO_4_ take for a reference. The photoluminescence (PL) spectra were performed by a RF-5301PC fluorescence spectrophotometer.

### Photocatalytic activity tests

The photocatalytic properties of the as-fabricated GO/BiOCl were assessed by visible-light degradation of RhB under room temperature (a 350-W Xe lamp with a cut-off filter, λ>420 nm). In the photocatalytic test, the GO/BiOCl photocatalyst was added into a breaker with 100 mL 2.5 mg/L RhB aqueous solution. In order to establish the adsorption-desorption equilibrium, the mixture was stirred for 30 min in dark before the start of photocatalytic experiment. At given intervals, 5 mL suspension was collected and examined by the UV-vis spectrophotometer.

### Photoelectrochemical measurements

The transient photocurrent responses and electrochemical impedance spectroscopy (EIS) measurements were measured in an electrochemical workstation (SP-150, France). The platinum wire was used as the counter electrode and the saturated Ag/AgCl electrode used as the reference electrode. The photo electrochemical experiments were conducted in 0.1 M Na_2_SO_4_ electrolyte solution.

## Results and discussion

### Characterization of the GO/BiOCL

**Figure 1:**
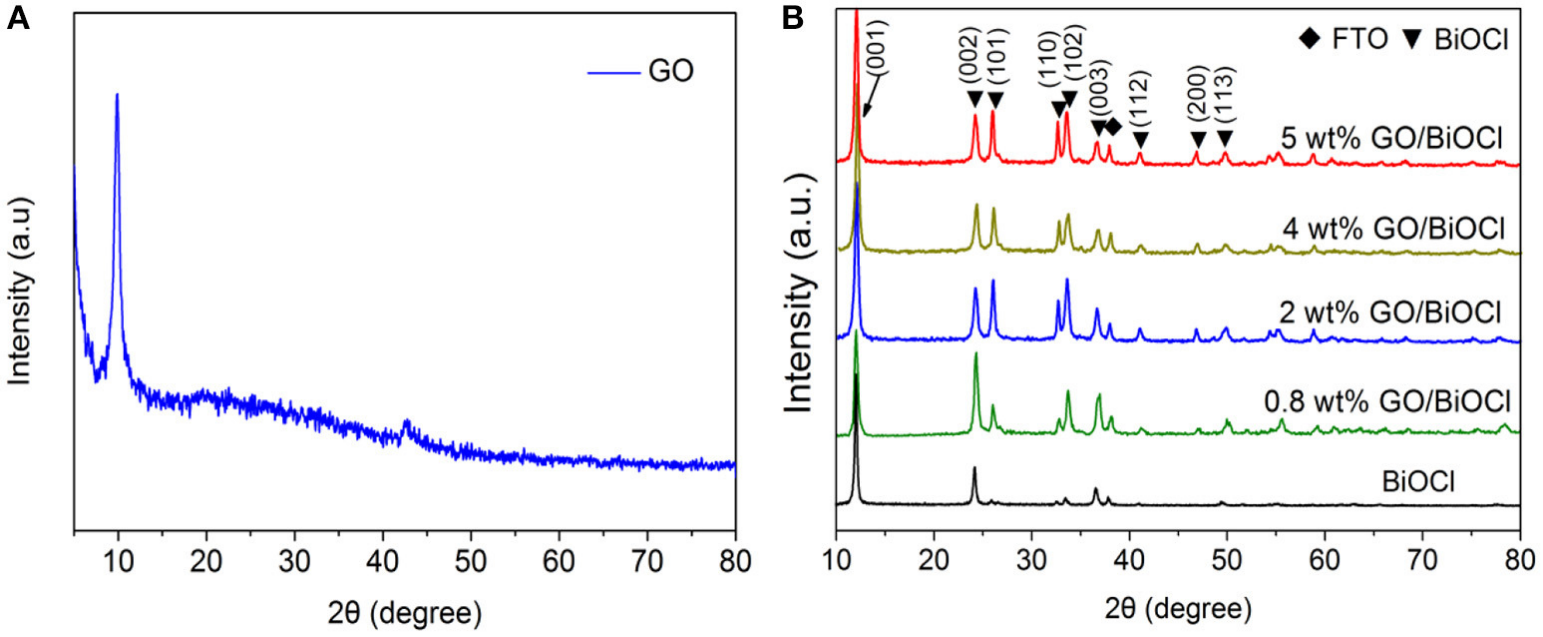
XRD patterns of **(A)** GO, **(B)** pure BiOCl and GO/BiOCl with different GO contents.

XRD patterns of GO, pure BiOCl and the as-prepared GO/BiOCl with different GO contents was shown in Figure 1. In Figure 1A, the peak at 10.6° belonged to GO, which was agreed with the reported results (Shin et al., 2009). The diffraction peaks of BiOCl were identical to those of tetragonal BiOCl, which suggested the high purity (Figure 1B). However, peaks of GO in the GO/BiOCl samples could not be observed, which owes to the low GO content in GO/BiOCl (Du et al., 2011). It was noticed that the peaks of BiOCl for GO/BiOCl samples obviously increased, suggesting that BiOCl grown on GO adopted a better crystallinity. The similar phenomenon was also observed in the previous report (Gao et al., 2012).

**Figure 2:**
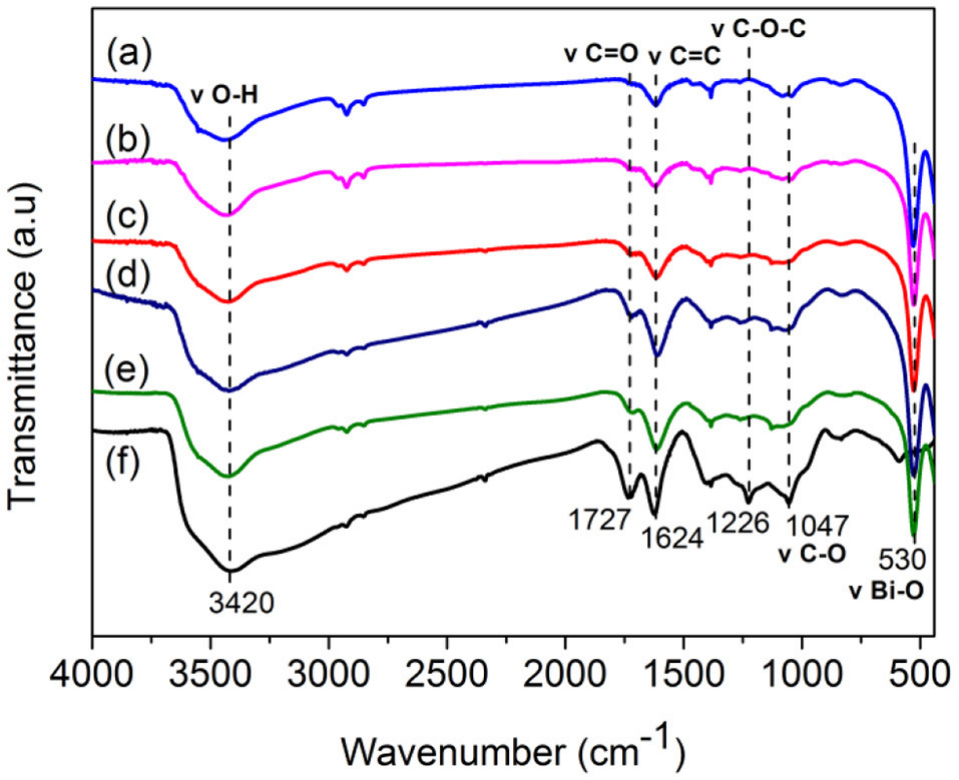
FT-IR spectra of **(a)** BiOCl; **(b)** 0.8 wt% GO/BiOCl; **(c)** 2 wt% GO/BiOCl, **(d)** 4 wt% GO/BiOCl; **(e)** 5 wt% GO/BiOCl; **(f)** GO.

The FT-IR spectra of BiOCl, GO and GO/BiOCl with different GO contents were shown in Figure 2. The characteristic peaks of oxygen-containing functional groups of GO were observed, such as C = O stretching vibration at 1,727 cm^−1^ from carboxyl or carbonyl groups, C = C skeletal vibration at 1,624 cm^−1^ from un-oxidized graphitic domains, epoxide C—O—C, or phenolic C—O—H stretching at 1,226 cm^−1^, and C—O stretching vibration at 1,047 cm^−1^ from epoxy groups (Szabó et al., 2006; Fu and Wang, 2011; Chen et al., 2012; Wang P. et al., 2013). The peak at 3,420 cm^−1^ was ascribed to the absorption of water or O—H groups (Liu et al., 2013). The GO/BiOCl samples had similar spectrum of GO but with lower peak intensity, which indicated the partial reduction of GO (Chen et al., 2012). In all the GO/BiOCl samples, the prominent peaks at about 530 cm^−1^ corresponded to Bi—O vibration (Chou et al., 2013). The appearance of the broad absorption at 1,150 cm^−1^ referring to Bi—C vibration, suggested a chemical bonding between GO and BiOCl (Tu et al., 2012).

**Figure 3:**
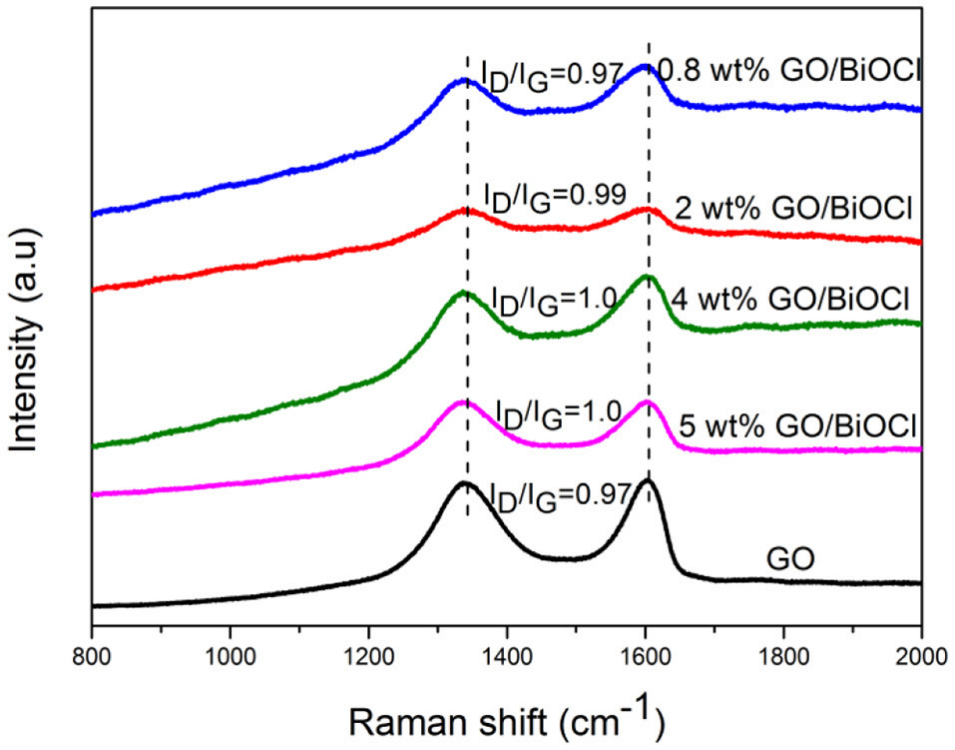
Raman spectra of GO and GO/BiOCl composites.

Raman spectroscopy was an important technique to study the structural properties of crystal and carbon materials. Figure 3 showed the Raman spectra of GO and GO/BiOCl composites. As shown, GO displayed two characteristic peaks at around 1,340 and 1,600 cm^−1^, which were ascribed to the D band and G band of graphite structures. However, in the GO/BiOCl composites, the D band shifted to around 1,334 cm^−1^ and G band moved to around 1,602 cm^−1^, which could be attributed to the chemical interaction between GO and BiOCl (Hu et al., 2014). I_D_/I_G_ ratio was used to represent the degree of graphitization. The I_D_/I_G_ intensity of GO was 0.97, but the I_D_/I_G_ intensity ratio increased with the increase of GO content, which suggested the reduction of GO during the procedure (Liu et al., 2014).

**Figure 4:**
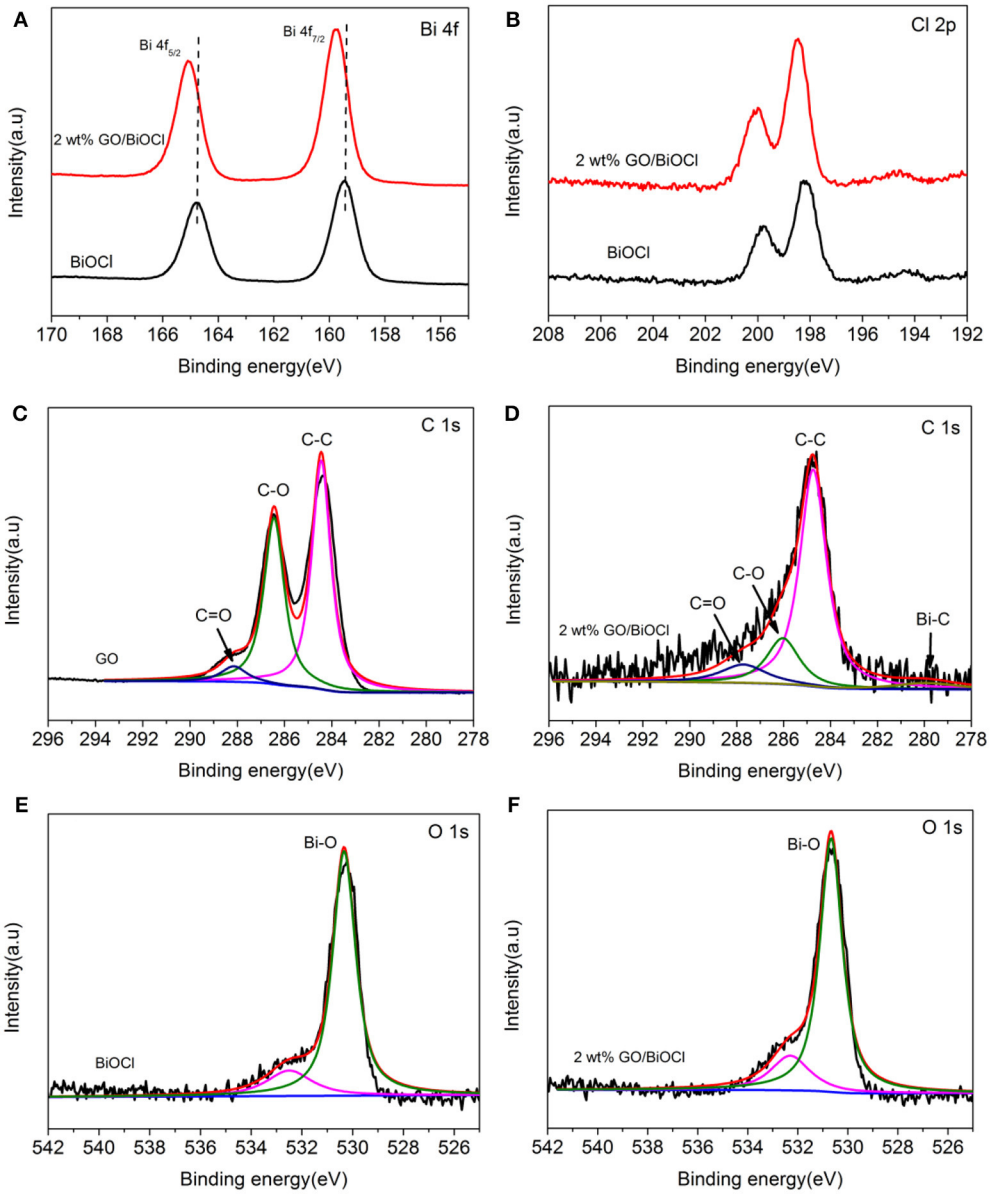
XPS spectra of BiOCl and 2 wt% GO/BiOCl: **(A)** Bi 4f; **(B)** Cl 2p; **(C)** C 1s of GO; **(D)** C 1s of 2 wt% GO/BiOCl; **(E)** O 1s of BiOCl; **(F)** O 1s of 2 wt% GO/BiOCl.

The XPS spectra provided the information for chemical state and surface properties of the 2 wt% GO/BiOCl film. According to Figure 4A, two strong peaks centered at 159.7 eV and 165.0 eV in 2 wt% GO/BiOCl were ascribed to Bi 4f_7/2_ and Bi 4f_5/2_, suggesting Bi^3+^ in the GO/BiOCl (Ai et al., 2011). The peaks of Bi 4f in the 2 wt% GO/BiOCl shifted slightly toward higher binding energies compared with pristine BiOCl, due to the strong interaction between BiOCl and GO (Tian L. et al., 2013). The Cl 2p of XPS spectra were displayed in Figure 4B. The peaks located at 198.4 eV and 199.9 eV, corresponded to Cl 2p_3/2_ and Cl 2p_1/2_, which were characteristics of Cl^−^in GO/BiOCl (Cheng et al., 2013). The XPS spectrum of C1s on GO was shown in Figure 4C. The peak at 284.7 eV was ascribed to C-C bond with sp^2^ orbital. The peaks located at 286.5 eV and 288.4 eV were attributed to the C-O and C = O suggesting the existence of oxygen-containing functional groups in the GO (Liu et al., 2014). In the spectrum of 2 wt% GO/BiOCl (Figure 4D), the peak of C-O and C = O showed lower intensities than those of GO, suggesting the partial reduction of GO, which corresponded to the FTIR spectra (Liu et al., 2014). Besides, a new peak at 281.7 eV appeared, which was related to carburetion, and referred to the existence of Bi-C in 2 wt% GO/BiOCl. The result was in accordance with the FTIR spectrum (Akhavan and Ghaderi, 2009). The O 1s region of XPS spectra for BiOCl and 2 wt% GO/BiOCl were depicted in Figures 4E,F. The peak at 532 eV could be ascribed to the Bi-O bond in [Bi_2_O_2_] slabs of BiOX layered structure. The peak centered at 530.3 eV was related to the hydroxyl groups or water molecules absorbed on the surface of the sample (Liu et al., 2012). The peak of hydroxyl in the 2 wt% GO/BiOCl showed higher intensities than that of BiOCl, indicating more oxygen-containing groups on the GO/BiOCl.

**Figure 5:**
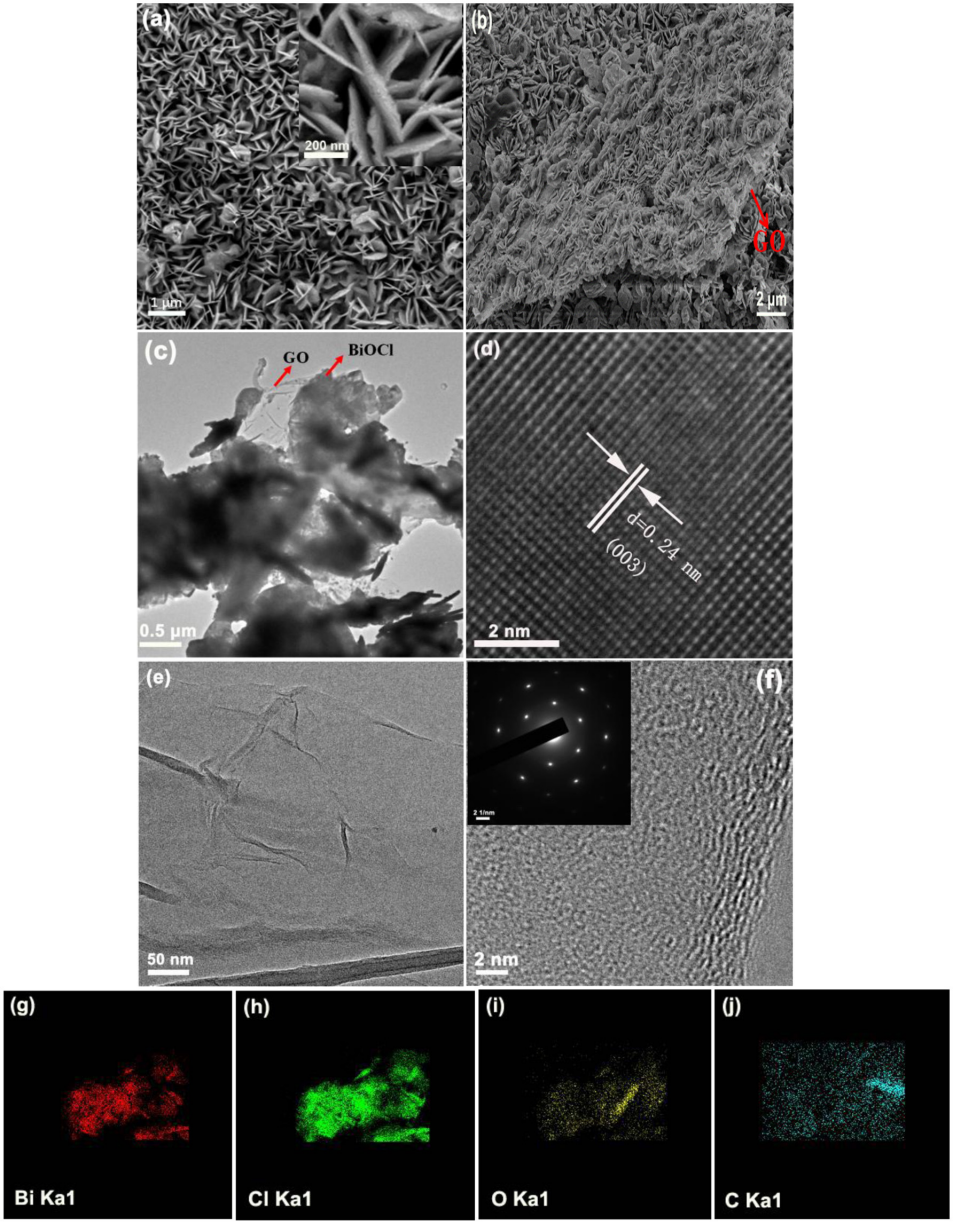
**(a,b)** FE-SEM image of BiOCl and 2 wt% GO/BiOCl; **(c)** TEM image of 2 wt% GO/BiOCl; **(d)** HRTEM image of BiOCl; **(e,f)** FE-SEM and HRTEM images of GO; **(g–j)** chemical element mapping data of 2 wt% GO/BiOCl.

The morphology and structure of 2 wt% GO/BiOCl were evaluated by SEM and TEM. Figure 5a showed the nanosheet-like morphology of the BiOCl with the size of 400–600 nm. Figure 5b showed that nanosheet-like BiOCl distribute uniformly onto the framework of GO in GO/BiOCl. Figure 5c depicted the TEM image of 2 wt% GO/BiOCl. As shown, the GO sheets were not very flat but displayed wrinkles. The structure of GO was shown in Figures 5e,f. A high-resolution TEM (HRTEM) image of BiOCl nanosheet (Figure 5d) exhibited the lattice spacing of 0.24 nm corresponding to (003) plane. As shown in Figures 5g–j, the signals of element Bi, Cl, O and C were clearly observed respectively, evidencing that the samples was GO/BiOCl.

### Nitrogen adsorption-desorption analysis

**Figure 6:**
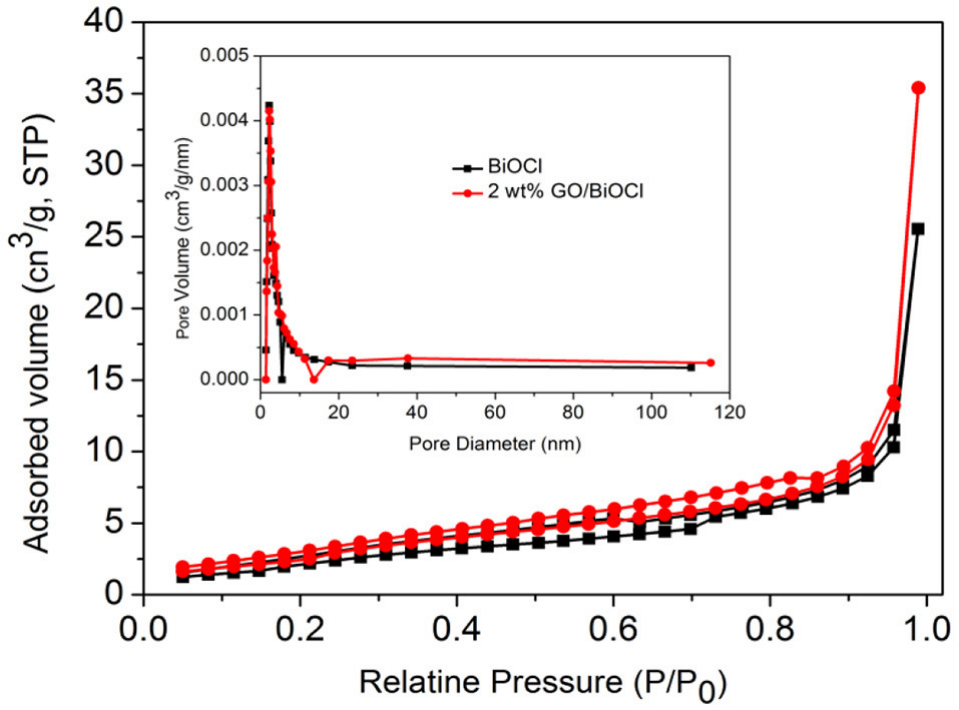
Nitrogen adsorption-desorption isotherm plot and corresponding pore-size distributions (inset) of BiOCl and 2 wt% GO/BiOCl.

Figure 6 presented the N_2_ adsorption-desorption isotherm and corresponding pore size distribution (PSD) cures of BiOCl and 2 wt% GO/BiOCl. Both of them were of type IV isotherms with a hysteresis loop within the range from 0.4 to 0.9 (*P/P*_0_), confirming the mesoporous structure. The BET surface areas of the composites were exhibited in Table 1. The *S*_BET_ of the GO/BiOCl composites were much larger than the pure BiOCl, indicating GO could improve the surface area of the GO/BiOCl composites, which was a significant factor leading to the enhanced photocatalytic activity. As shown in the inset of Figure 6, the pore sizes of the samples were found to be at about 2–11 nm.

**Table 1:** Summarized BET surface areas and catalytic properties of the photocatalysts.

**Sample**	***S*_BET_ (m^2^ g^−1^)**	***k* (h^−1^)**
BiOCl	3.49	0.24
GO	21.68	0.52
0.8 wt% GO/BiOCl	7.22	0.41
2 wt% GO/BiOCl	11.16	2.93
4 wt% GO/BiOCl	11.29	1.92
5 wt% GO/BiOCl	12.73	1.84

### Optical absorption properties

**Figure 7:**
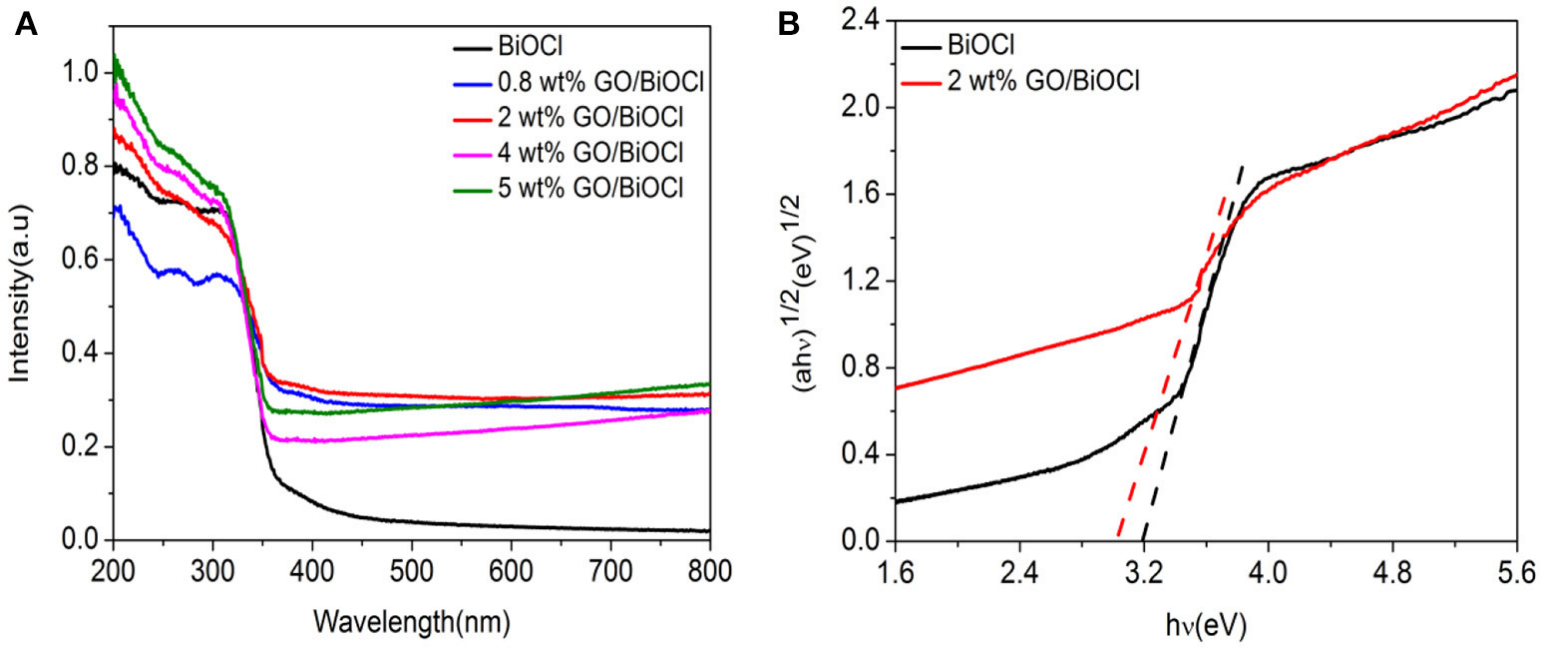
**(A)** UV-visdiffuse reflectance of the as-prepared samples; **(B)** the band gaps energies (Eg) of pure BiOCl and 2 wt% GO/BiOCl.

Figure 7 revealed the UV-vis DRS of pure BiOCl and GO/BiOCl composites. It was noticed that pure BiOCl showed absorption only in UV region. With the introduction of GO, the absorption intensity in the visible-light region of the GO/BiOCl samples was improved. This result indicated that GO played a major role in utilizing sunlight and worked as an electron reservoir to trap the electrons under irradiation (Wei et al., 2014), leading to better photocatalytic performance consequently. The Eg of the GO/BiOCl could be figured out according to previous report (Xie et al., 2013). As can be seen in Figure 7B, the band gap energy of pure BiOCl and 2 wt% GO/BiOCl were estimated to be 3.2 and 2.9 eV. It could be seen that the estimated band gap energy of the 2 wt% GO/BiOCl film was lower than that of pristine BiOCl, suggesting that hybridizing BiOCl with GO could enhance the optical absorption property of BiOCl in visible-light region.

### Photocatalytic properties

**Figure 8:**
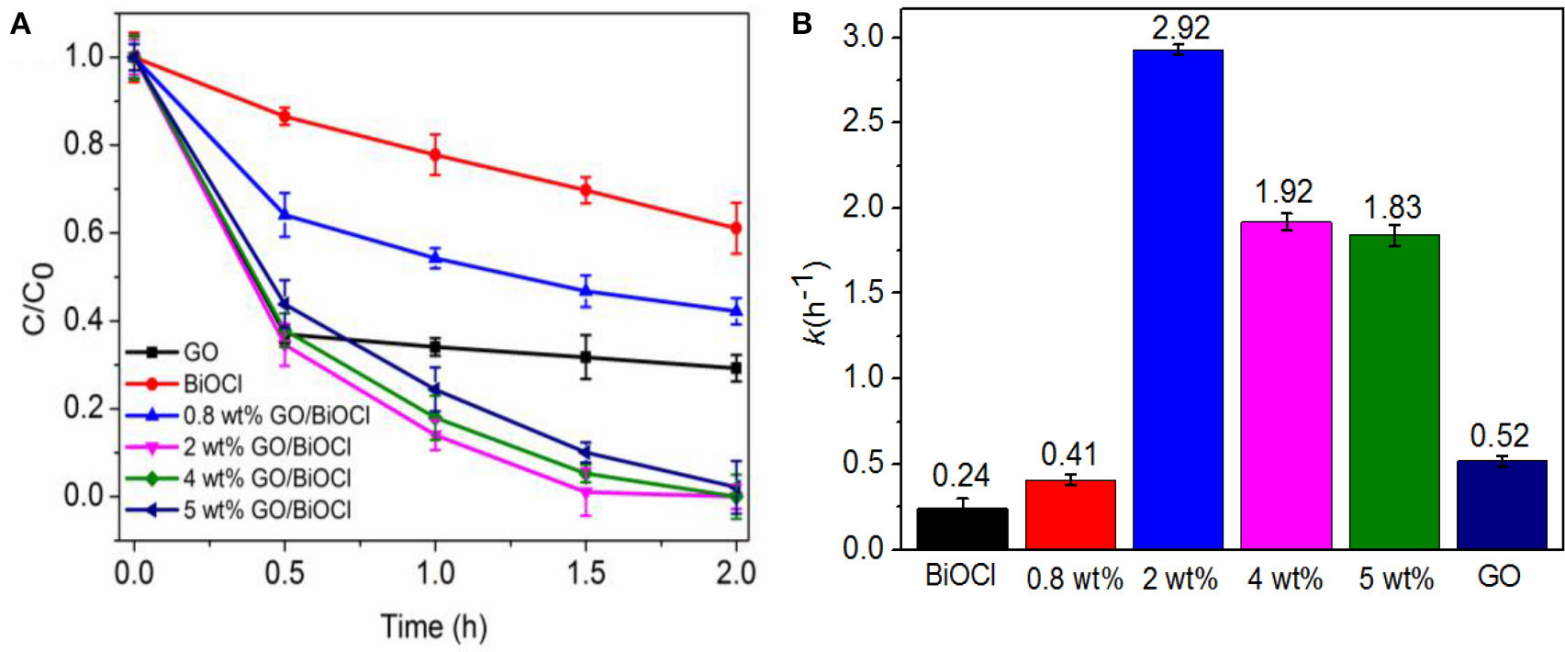
**(A)** Comparison of photocatalytic activities of the as-prepared samples on the degradation of RhB under visible-light irradiation (λ>420 nm); **(B)** Reaction kinetics of RhB degradation under visible light irradiation by pure BiOCl, GO and GO/BiOCl composites.

The photocatalytic performance of the as-fabricated GO/BiOCl was studied by visible-light degradation toward RhB in an aqueous solution. As can be seen in Figure 8A, GO/BiOCl displayed higher photodegradation efficiency compared to pure BiOCl. Along with the increase of GO content, the degradation efficiency of GO/BiOCl increased at first, but then decreased while the GO content was larger than 2 wt%. So the highest photocatalytic activity of GO/BiOCl was gained with the optimum content of GO located at 2 wt%, which led to 99% visible-light degradation of RhB within 1.5 h. The degradation rate constants (*k*, h^−1^) were calculated to be 0.24, 0.41, 2.93, 1.92, 1.84 and 0.52 h^−1^ for pure BiOCl, 0.8, 2, 4 wt%, 5 wt% GO/BiOCl and GO respectively (Figure 8B). It could be seen that all GO/BiOCl showed much higher *k*-values compared to pristine BiOCl, and 2 wt% GO/BiOCl was about 12 times that of pristine BiOCl. As GO content further increased, the photocatalytic activity of RhB degradation obviously decreased, suggesting that suitable content of the GO was important to improve the photocatalytic activity.

The photocatalytic activity was also compared with other film photocatalysts (Table 2). The as-prepared 2 wt% GO/BiOCl film had a higher photocatalytic activity than those photocatalysts, which indicated that it had great prospect for practical applications in degrading pollutants.

**Table 2:** Photocatalytic activities of various filmphotocatalysts on the degradation of RhB.

**Photocataltsts**	**Dye concentration (mg/L)**	**Photocatalytic activity**	**References**
**Films**			
TiO_2_	10	50% degraded within 5 h (UV-light)	Wang C. et al., 2012
BiOCl	1.0	52.5% degraded within 8 h (visible-light)	Liang et al., 2013
BiOBr	5.0	80% degraded within 8 h (visible-light)	Cuellar et al., 2015
ZnO:I/TiO_2_	2.4	97% degraded within 6 h (visible-light)	Wang et al., 2015
Bi_2_WO_6_	5.0	53% degraded within 12 h (visible-light)	Zhao et al., 2007
Bi_2_O(OH)_2_SO_4_	1.0	92% degraded within 7 h (visible-light)	Zhang et al., 2015
GO/BiOCl	2.5	99% degraded within 1.5 h (visible-light)	This work

**Figure 9:**
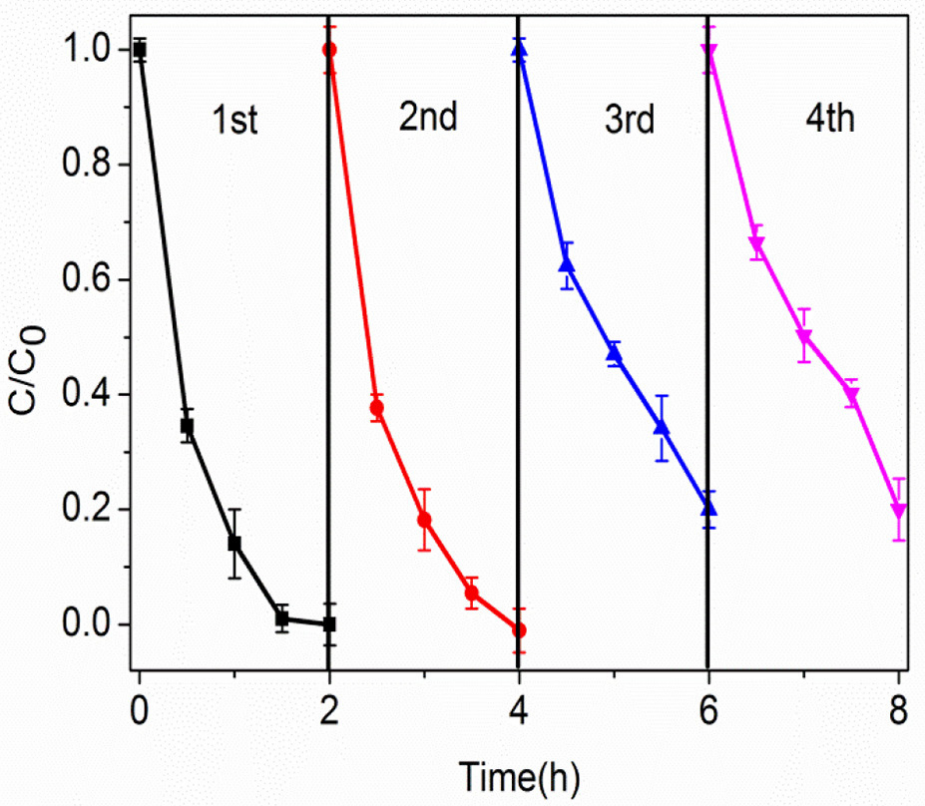
Recycling tests of 2 wt% GO/BiOCl for the degradation of RhB under visible light irradiation.

The stability and regeneration of the photocatalyst are important to the practical applications. Due to immobilization, the GO/BiOCl samples could be directly separated from the aqueous solution for next recycle. Figure 9 depicted the photocatalytic activities of 2 wt% GO/BiOCl for degradation of RhB within four cycles. It could be seen that the visible-light photodegradation efficiencies of RhB can reach 80%. These results suggested that the GO/BiOCl had excellent stability and regeneration and could be used as an effective photocatalyst in practical application.

### Possible photocatalytic mechanism of GO/BiOCL

**Figure 10:**
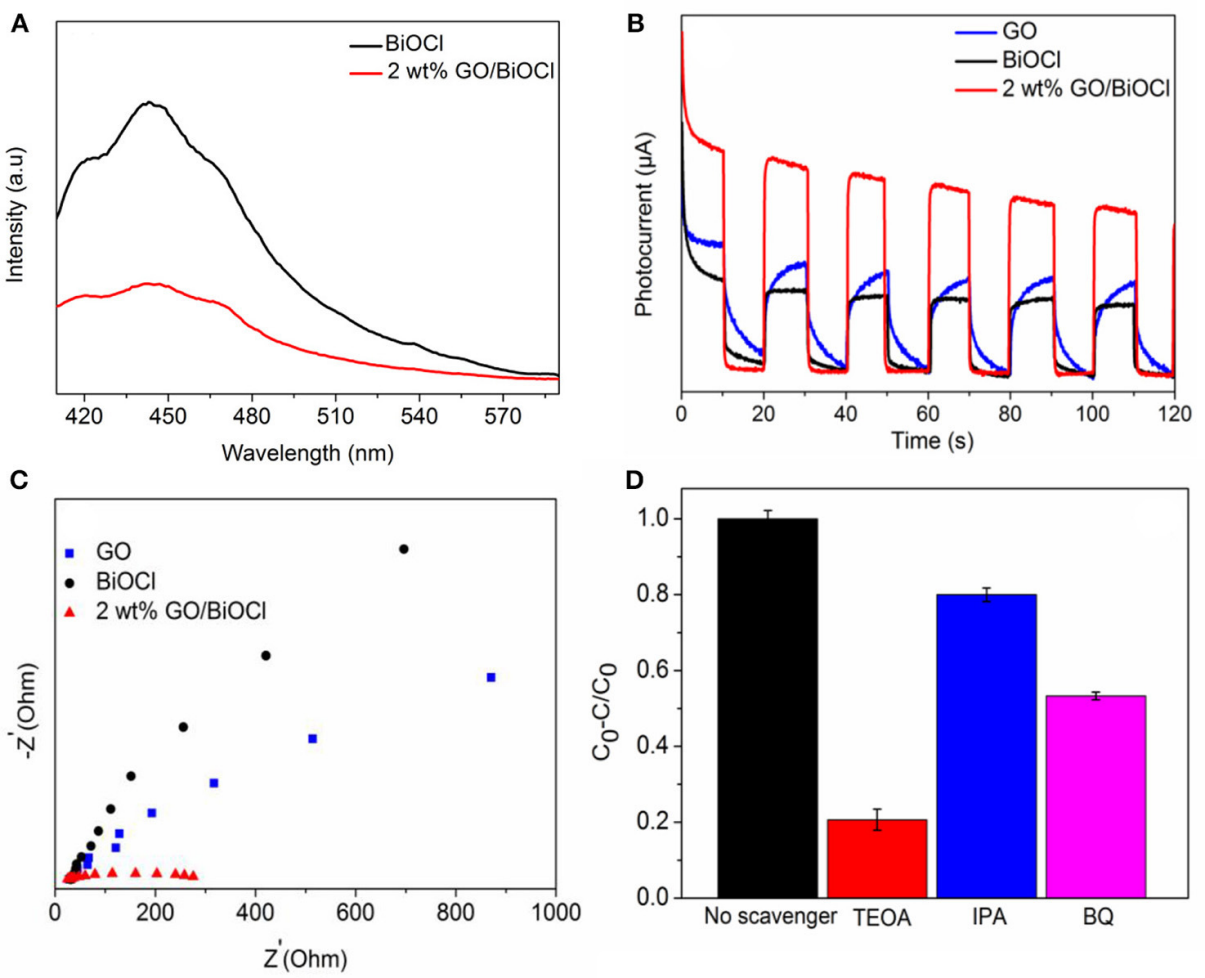
**(A)** PL spectra of pure BiOCl and 2 wt% GO/BiOCl; **(B)** transient photocurrent response of pure BiOCl, GO and 2 wt% GO/BiOCl; **(C)** electrochemical impedance spectra of pure BiOCl, GO and 2 wt% GO/BiOCl; **(D)** the effect of reactive species in the photogradation process of RhB over 2 wt% GO/BiOCl.

PL emission spectra have also been regarded as an efficient way to discuss the charge transportation and separation of photocatalysts. As we know, a lower PL intensity correlated with a higher separation efficiency of electron-hole pairs (Tian G. et al., 2013). As was depicted in Figure 10A, the emission spectra of pristine BiOCl and 2 wt% GO/BiOCl were similar, but the intensity for 2 wt% GO/BiOCl was lower than pure BiOCl, which implied that 2 wt% GO/BiOCl had a lower recombination rate of photogenerated charge carriers resulting in enhanced photocatalytic activities.

The transient photocurrent responses of BiOCl, GO and 2 wt% GO/BiOCl under visible light irradiation in on-off cycles were investigated. In Figure 10B, 2 wt% GO/BiOCl exhibited a significantly increased photocurrent density, which was about much stronger than that of BiOCl and GO, implying that the recombination of photogenerated carriers in 2 wt% GO/BiOCl composite was suppressed (Fan et al., 2016; Feng Y. et al., 2017). Figure 10C depicted the corresponding EIS of pure BiOCl, GO and 2 wt% GO/BiOCl. 2 wt% GO/BiOCl exhibited the smallest diameter of the Nyquist circle, demonstrating that the transfer efficiency of photoinduced carriers was improved in 2 wt% GO/BiOCl (Hao et al., 2017). All the PL, transient photocurrent response and EIS results suggested that the separation and transfer efficiency of electron-hole pairs were substantially improved by the addition of GO.

In order to further reveal the photocatalytic mechanism of 2 wt% GO/BiOCl in the photocatalytic degradation process, the trapping experiments of 2 wt% GO/BiOCl were conducted using different scavengers. As shown in Figure 10D, isopropanol (IPA), benzoquinone (BQ), and triethanolamine (TEOA) were used as the scavenger for hydroxyl radicals, superoxide radicals and holes (Li et al., 2017a,b,c). On one hand, the photodegradation efficiency of RhB was slightly decreased by adding IPA, which indicated that ·OH was not a major active species. On the other hand, with the addition of benzoquinone or triethanolamine into the system, the photodegradation of RhB decreased obviously suggesting that ·${\text{O}}_{2}^{-}$ radicals and h^+^ played a dominant part in the visible-light degradation process. This was different from the conclusions that e^−^ was the main active species for GO/BiOX composites in other relative reports.

**Figure 11:**
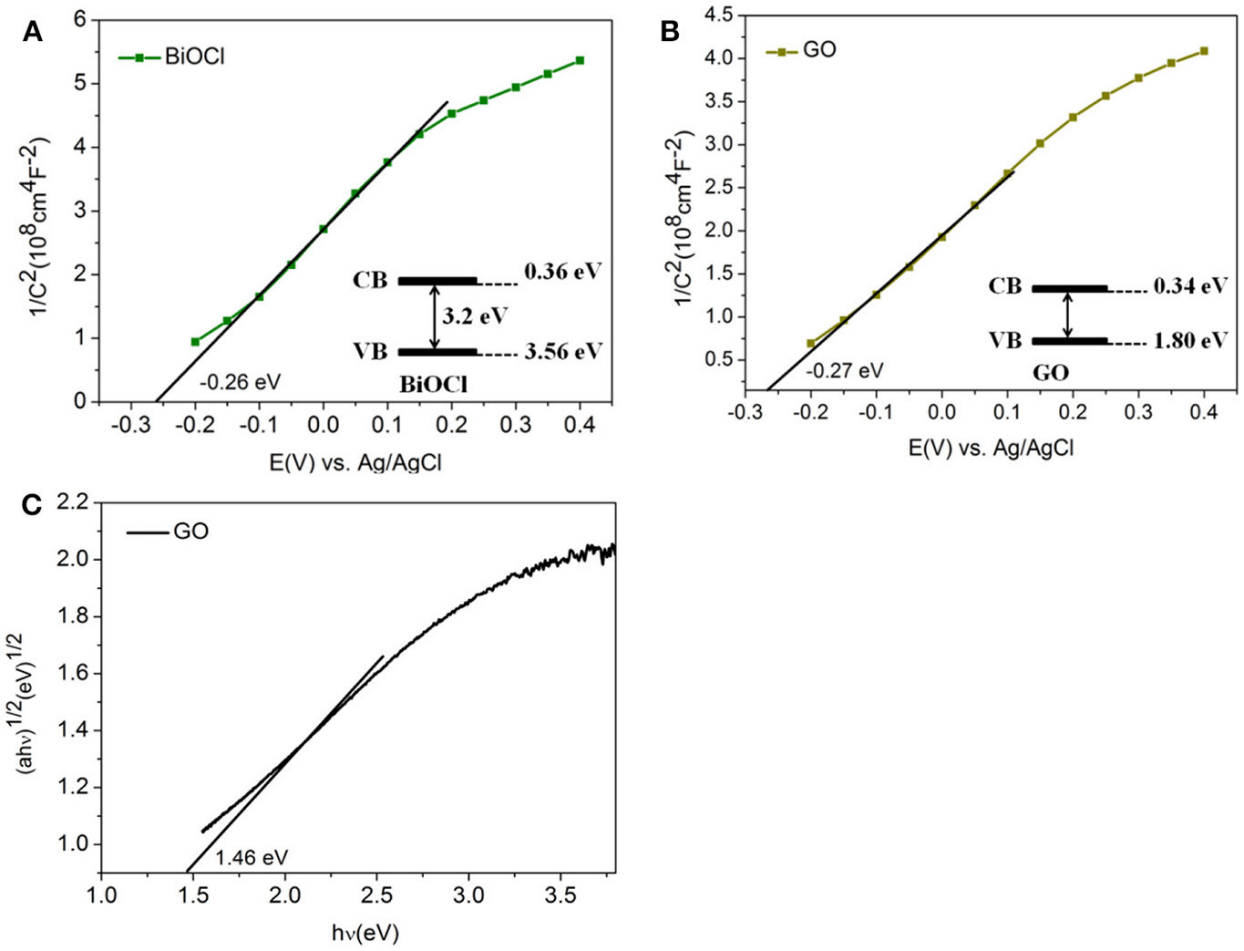
**(A)** Mott-Schottky plots of BiOCl; **(B)** Mott-Schottky plots of GO; **(C)** band gaps energies (Eg) of GO.

Figure 11 showed the Mott-Schottlyplots for BiOCl and GO. The positive slope of the C^−2^-E indicated the expected n-type BiOCl and GO of the film (Kong, 2008; Weng et al., 2013). The extrapolations of the Mott-Schottly plots provided a good approximation of the flat band potential, the value of which were about −0.26 and −0.27 eV for BiOCl and GO respectively. The measured potentials could be converted to the reversible hydrogen electrode scale *via* the Nernst equation (Yang G. et al., 2017; Yang J. et al., 2017), so the conduction band edges were calculated to be 0.36 and 0.34 eV. Based on the values of conduction band and the band gap, the valence band positions could be calculated to be 3.56 eV and 1.80 eV *via E*_*CB*_ = *E*_*VB*_ − *Eg* (Duo et al., 2015), in which *Eg* was about 3.2 eV (Figure 7B) and 1.46 eV (Figure 11A) for BiOCl and GO.

**Figure 12:**
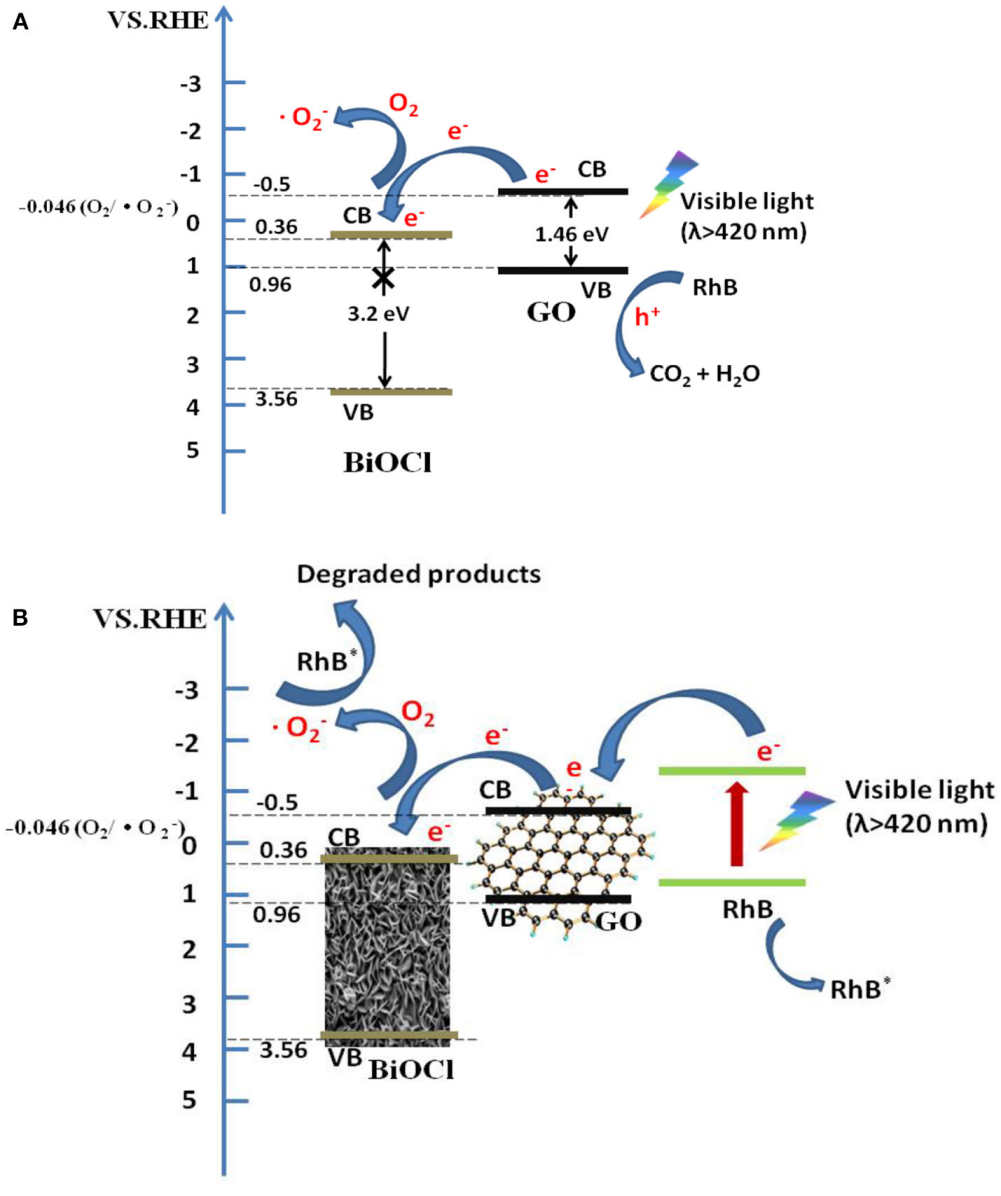
Schematic illustration of the proposed photocatalytic mechanism for GO/BiOCl composites degrading RhB under visible light irradiation: photodegradation **(A)** and photosensitization **(B)**.

According to the above results, a new photocatalytic mechanism of GO/BiOCl nanocomposite including photodegradation and photosensitization at the same time, which was totally different from relevant reports, could be proposed as follows. (1) Holes played a dominant part in the visible-light degrading RhB process, suggesting that photodegradation was an important mechanism. Under visible light irradiation, GO was inspired and gave photo-generated electrons and holes. Electrons flew into the CB of BiOCl, which reacted with O_2_ to generate ·${\text{O}}_{2}^{-}$. ·${\text{O}}_{2}^{-}$ and the holes on the VB of GO degraded the contaminant (Figure 12A). (2) Salicylic acid was slightly degraded under visible light irradiation (Figure S1), indicating photosensitization was another important mechanism. Under visible light irradiation, RhB was excited to produce electrons, which flew into the CB of GO, leaving the activated RhB molecules (RhB^*^). And the electrons on the CB of GO would further transfer to BiOCl, which reacted with O_2_ to generate ·${\text{O}}_{2}^{-}$, and degraded RhB^*^ directly (Figure 12B). During the photosensitization process, holes are not detected to be a major active species.

## Conclusion

In summary, GO/BiOCl composite films were successfully prepared *via* a facile spread coating method. The GO/BiOCl films especially 2 wt% GO/BiOCl exhibited a much higher photocatalytic activity compared with pristine BiOCl and many other film photocatalysts, which could be mainly ascribed to the improved light adsorption and separation of photoinduced electrons and holes both resulting from GO. A new photocatalytic mechanism totally different from relevant reports, was revealed to include photodegradation and photosensitization at the same time. In addition, their recycle was much easier and realizable owing to the immobilization of GO/BiOCl on FTO, and excellent recyclability under visible light was obtained. This work could provide a new way to developing novel composite film photocatalysts and studying new photocatalytic mechanisms for similar GO/BiOX nanocomposites.

## Author contributions

WL conceived and designed the experiments, performed the experiments, analyzed the data, and drafted the manuscript. WL and YiZ revised the manuscript. XY and YuZ contributed significantly to the manuscript preparation.

### Conflict of interest statement

The authors declare that the research was conducted in the absence of any commercial or financial relationships that could be construed as a potential conflict of interest.
