# Supplementary material for: Graphene Oxide/BiOCl Nanocomposite Films as Efficient Visible Light Photocatalysts
Source: Front Chem. 2018 Jul 24;6:274. doi: 10.3389/fchem.2018.00274 (PMC6066524; doi:10.3389/fchem.2018.00274)
Supplement: Supplementary file 1 [file Image_1.pdf]

Supplementary material

Graphene oxide/BiOCl nanocomposite films as efficient visible light photocatalysts

WeitianLin[a], Xiang Yu[b], Yi Zhu[a,*], Yuanming Zhang[a]

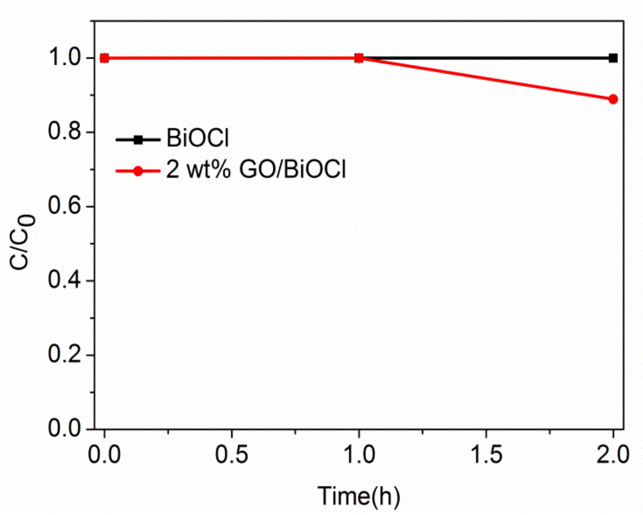

Fig. S1 Photodegradation of salicylic acid by 2 wt % GO/BiOCl and BiOCl under visible-light ($\lambda > 420$ nm) irradiation
